# On a class of N-dimensional anisotropic Sobolev inequalities

**DOI:** 10.1186/s13660-018-1754-3

**Published:** 2018-07-05

**Authors:** Lirong Huang, Eugenio Rocha

**Affiliations:** 1 College of Mathematics and Physics, Fujian Jiangxia University, Fuzhou, P.R. China; 2 Department of Mathematics, University of Aveiro, Aveiro, Portugal

**Keywords:** 35J20, 35A30, Anisotropic Sobolev inequality, Smallest constant, Minimal action solution

## Abstract

In this paper, we study the smallest constant *α* in the anisotropic Sobolev inequality of the form

$$\Vert u \Vert _{p}^{p} \leq \alpha \Vert u \Vert _{2}^{\frac{2(2N-1)+(3-2N)p}{2}} \Vert u_{x} \Vert _{2}^{\frac {N(p-2)}{2}} \prod_{k=1}^{N-1} \bigl\Vert D_{x}^{-1}\partial_{y_{k}}u \bigr\Vert _{2}^{\frac{p-2}{2}} $$

and the smallest constant *β* in the inequality

$$\Vert u \Vert _{p_{*}}^{p_{*}} \leq\beta \Vert u_{x} \Vert _{2}^{\frac{2N}{2N-3}} \prod _{k=1}^{N-1} \bigl\Vert D_{x}^{-1} \partial_{y_{k}}u \bigr\Vert _{2}^{\frac{2}{2N-3}}, $$

where $V := (x, y_{1}, \ldots, y_{N-1})\in\mathbb{R}^{N}$ with $N\geq 3$ and $2 < p < p_{*} = {\frac{2(2N-1)}{2N-3}}$. These constants are characterized by variational methods and scaling techniques. The techniques used here seem to have independent interests.

## Introduction

Let $N\geq2$ and $2 < p \leq p_{*} := {{2(2N-1)}\over {2N-3}}$. A classical inequality [1, p. 323] states: there is a positive constant $C > 0$ such that, for any $f \in C_{0}^{\infty}(\mathbb{R}^{N})$,

1.1 $$ \begin{aligned}[b] \int_{\mathbb{R}^{N}} \vert f_{x} \vert ^{p} \,dV \leq{}& C \biggl( \int_{\mathbb{R}^{N}} \vert f_{x} \vert ^{2}\,dV \biggr)^{{2(2N-1)+(3-2N)p}\over {4}} \biggl( \int_{\mathbb{R}^{N}} \vert f_{xx} \vert ^{2}\,dV \biggr)^{{N(p-2)\over 4}} \\ &{}\times \prod_{k=1}^{N-1} \biggl( \int_{\mathbb{R}^{N}} \vert \partial_{y_{k}}f \vert ^{2}\,dV \biggr)^{{p-2}\over 4}, \end{aligned} $$

where $V := (x,y)\in\mathbb{R}^{N}$ and $y = (y_{1}, y_{2}, \ldots, y_{N-1})\in\mathbb{R}^{N-1}$. The purpose of the present paper is to characterize the smallest (sharp) positive constant *C* of (1.1) (see Theorem 2.8 and Theorem 3.8) and the related equations (see (2.1) and (3.2)).

Two special cases of (1.1) have been used to study the solitary waves of the generalized Kadomtsev–Petviashvili equation. For example, when $N = 2$ (at this moment, $V:= (x,y_{1})\in \mathbb{R}^{2}$ ) and $2 < p < 6$, (1.1) in the form

1.2 $$ \int_{\mathbb{R}^{2}} \vert u \vert ^{p}\,dV \leq C \biggl( \int_{\mathbb{R}^{2}} u_{x}^{2}\,dV \biggr)^{{p-2}\over 2} \biggl( \int_{\mathbb{R}^{2}} \bigl(D_{x}^{-1} \partial_{y_{1}}u\bigr)^{2}\,dV \biggr)^{{p-2}\over 4} \biggl( \int_{\mathbb{R}^{2}} u^{2}\,dV \biggr)^{{6-p}\over 4} $$

has been used to study the following generalized Kadomtsev–Petviashvili I equation:

1.3 $$ \varphi_{t} + \varphi_{xxx} + \varphi^{p-2}\varphi_{x} = D_{x}^{-1} \varphi_{y_{1}y_{1}},\quad(x,y_{1})\in \mathbb{R}^{2}, t > 0. $$

de Bouard et al. [6, 7] proved that (1.3) had a solitary wave solution for $2 < p < 6$ and (1.3) did not possess any solitary waves if $p \geq6$. Stability of solitary wave of (1.3) has been studied in [9] in which (1.2) has played an important role. Chen et al. [4] also used (1.2) to study the Cauchy problem of solutions to the 2-dimensional generalized Kadomtsev–Petviashvili I equation, generalized rotation-modified Kadomtsev–Petviashvili equation and generalized Kadomtsev–Petviashvili coupled with Benjamin–Ono equation.

When $N = 3$ (at this moment, $V:= (x,y_{1},y_{2})\in\mathbb{R}^{3}$), de Bouard et al. [6, 7] used (1.1) to prove that if $p \geq{10\over 3}$ then the following equation:

1.4 $$ -u + u_{xx} + u^{p-1} = D_{x}^{-2}u_{y_{1}y_{1}} + D_{x}^{-2}u_{y_{2}y_{2}},\quad u \neq0, $$

had no solutions in $Y(3)$, where $Y(3)$ is the closure of $\partial_{x}(C_{0}^{\infty}(\mathbb{R}^{3}))$ under the norm

$$\Vert u \Vert ^{2}_{Y(3)} = \int_{\mathbb{R}^{3}} \bigl(u_{x}^{2} + \bigl\vert D_{x}^{-1}\partial _{y_{1}}u \bigr\vert ^{2} + \bigl\vert D_{x}^{-1}\partial_{y_{2}}u \bigr\vert ^{2} + \vert u \vert ^{2} \bigr)\,dV. $$

Here we define $D_{x}^{-1}$, $D_{x}^{-2}$ by

$$D_{x}^{-1}h(x,y) = \int_{-\infty}^{x}h(s,y)\,ds,\qquad D_{x}^{-2}h = D_{x}^{-1}\bigl(D_{x}^{-1}h\bigr). $$

While for $2 < p < {10\over 3}$, (1.4) had at least one nonzero solution in $Y(3)$. Observing this previous work, $p_{*} = 6$ (when $N = 2$) and $p_{*} = {10\over 3}$ (when $N = 3$) seem to be a critical nonlinear exponent, which shares some properties similar to the critical Sobolev exponent $2^{*} = 2N/(N-2)$ ( $N\geq3$) in the study of semilinear elliptic equations. Recall that the best constant $C_{S}$ in the Sobolev inequality $\|u\|_{L^{2^{\ast}}}^{2} \leq C_{S} \|\nabla u\|_{L^{2}}^{2}$ is well-known and has been used extensively. But for the smallest constant *C* in (1.1), few results are known. When $N =2$ and $2 < p < 6$, the smallest constant *C* of (1.2) and its applications has been studied in [4]. When $N = 2$ and $p= 6$, the characterization of the smallest constant *C* of (1.2) and its related properties were studied in [5].

In the present paper, we are interested in the characterization of the smallest constant *C* of (1.1) in the case of $N\geq3$. According to the value of $2 < p < p_{*}$ and $p = p_{*} = 2(2N-1)/(2N-3)$, the studies were divided into two parts. In the first part, we study (1.1) in the case of $2 < p < p_{*}$. At this time, (1.1) is written as the following form:

1.5 $$ \begin{aligned}[b] \int_{\mathbb{R}^{N}} \vert u \vert ^{p}\,dV \leq{}& \alpha \biggl( \int_{\mathbb{R}^{N}} \vert u \vert ^{2}\,dV \biggr)^{{2(2N-1)+(3-2N)p}\over {4}} \biggl( \int_{\mathbb{R}^{N}} \vert u_{x} \vert ^{2}\,dV \biggr)^{{N(p-2)\over 4}} \\ & {}\times \prod_{k=1}^{N-1} \biggl( \int_{\mathbb{R}^{N}} \bigl\vert D_{x}^{-1} \partial_{y_{k}}u \bigr\vert ^{2}\, dV \biggr)^{{p-2}\over 4}, \end{aligned} $$

where $u\in Y_{1}$ and $Y_{1}$ is the closure of $\partial_{x}(C_{0}^{\infty}(\mathbb{R}^{N}))$ under the norm

$$\Vert u \Vert ^{2}_{Y_{1}} := \int_{\mathbb{R}^{N}} \bigl(u_{x}^{2} + \bigl\vert D_{x}^{-1}\nabla_{y} u \bigr\vert ^{2} + \vert u \vert ^{2} \bigr)\,dV. $$

As before and from now on, $y = (y_{1}, \ldots, y_{N-1})$,

$$\nabla_{y} = \biggl({\partial\over {\partial y_{1}}}, \ldots, {\partial\over {\partial y_{N-1}}} \biggr),\qquad \bigl\vert D_{x}^{-1} \nabla_{y}u \bigr\vert ^{2} = \sum _{k=1}^{N-1} \bigl\vert D_{x}^{-1} \partial_{y_{k}}u \bigr\vert ^{2}\quad \hbox{and}\quad \triangle_{y} = \sum_{k=1}^{N-1} {{\partial^{2}}\over {\partial y_{k}}}. $$

The main result of this part is to prove that the smallest constant *α* can be represented by *N*, *p* and a minimal action solution of

$$-u + u_{xx} + \vert u \vert ^{p-2}u = D_{x}^{-2}\triangle_{y}u,\quad u\neq0, u\in Y_{1}. $$

For details, see Theorem 2.5 and Theorem 2.8.

In the second part, we treat (1.1) in the case of $p = p_{*}$. In this case, (1.1) is written as

1.6 $$ \int_{\mathbb{R}^{N}} \vert u \vert ^{p_{*}}\,dV \leq\beta \biggl( \int_{\mathbb{R}^{N}} \vert u_{x} \vert ^{2}\,dV \biggr)^{N\over {2N-3}} \prod_{k=1}^{N-1} \biggl( \int_{\mathbb{R}^{N}} \bigl\vert D_{x}^{-1} \partial_{y_{k}}u \bigr\vert ^{2}\, dV \biggr)^{1\over {2N-3}}, $$

where $u\in Y_{0}$ and $Y_{0}$ is the closure of $\partial_{x}(C_{0}^{\infty}(\mathbb{R}^{N}))$ under the norm

$$\Vert u \Vert ^{2}_{Y_{0}} := \int_{\mathbb{R}^{N}} \bigl(u_{x}^{2} + \bigl\vert D_{x}^{-1}\nabla_{y} u \bigr\vert ^{2} \bigr)\,dV. $$

The main results of this part are Theorem 3.5 and Theorem 3.8.

The estimate of the smallest constants *α* and *β* is based on variational methods and scaling techniques. Recall that Weinstein [10] used variational methods to estimate the constant $C_{G}$ in the Gagliardo–Nirenberg interpolation inequality [3],

$$\int_{\mathbb{R}^{N}} \vert u \vert ^{q+1}\,dz \leq C_{G} \biggl( \int_{\mathbb{R}^{N}} \vert \nabla u \vert ^{2}\,dz \biggr)^{{N(q-1)}\over 4} \biggl( \int_{\mathbb{R}^{N}} \vert u \vert ^{2}\,dz \biggr)^{{2(q+1)-N(q-1)}\over 4},\quad u\in W^{1,2}\bigl(\mathbb{R}^{N} \bigr). $$

This $C_{G}$ was estimated directly by studying the following minimization problem:

$$C_{G}^{-1} = \inf \biggl\{ {{(\int_{\mathbb{R}^{N}} \vert \nabla u \vert ^{2}\,dz)^{{N(q-1)}\over 4}(\int_{\mathbb{R}^{N}} \vert u \vert ^{2}\,dz)^{{2(q+1)-N(q-1)}\over 4}}\over {\int_{\mathbb{R}^{N}} \vert u \vert ^{q+1}\,dz}} : u\in W^{1,2}\bigl(\mathbb{R}^{N}\bigr)\backslash\{0\} \biggr\} , $$

due to the compactness embedding of

$$W_{\mathrm{radial}}^{1,2}\bigl(\mathbb{R}^{N}\bigr) \hookrightarrow L^{q+1}\bigl(\mathbb {R}^{N}\bigr)\quad\hbox{for }1 < q < 2^{\ast}-1, $$

where

$$W_{\mathrm{radial}}^{1,2}\bigl(\mathbb{R}^{N}\bigr) = \bigl\{ u \in W^{1,2}\bigl(\mathbb{R}^{N}\bigr) : u(x) = u\bigl( \vert x \vert \bigr) \bigr\} , $$

$2^{\ast}= 2N/(N-2)$ for $N\geq3$ and $2^{\ast}= +\infty$ for $N = 2$. Weinstein [10] managed to prove the best constant $C_{G}$ for $N\geq2$ because the above compactness embedding holds only for $N\geq2$. However, in the process of studying the best constant *α* (respectively, *β*), we cannot use the methods of Weinstein [10] because we are facing anisotropic Sobolev spaces $Y_{1}$ (respectively, $Y_{0}$). In the present paper, we introduce a new method. The detailed strategy contains three steps, which are given in the next section; and it may have independent interest. In fact, we believe that it can be used to study the smallest constant of other kind of inequalities.

This paper is organized as follows. In Sect. 2, we study the constant *α*, meanwhile we explain the strategy in detail. In Sect. 3, we use this method to study the smallest constant *β* under some additional analytic techniques.

### Notations

Throughout this paper, all integrals are taken over $\mathbb{R}^{N}$ unless stated otherwise. A function *u* defined on $\mathbb{R}^{N}$ is always real-valued. $\|\cdot\|_{q}$ denotes the $L^{q}$ norm in $L^{q}(\mathbb{R}^{N})$.

## The smallest constant *α*

In this section, we always assume that $2 < p < p_{*} := {{2(2N-1)}\over {2N-3}}$. We introduce a new strategy to estimate *α* in (1.5). It contains three steps and hence we divide this section into three subsections.

### Minimal action solutions

In this subsection, we prove the existence of the minimal action solutions of the following equation:

2.1 $$ -u + u_{xx} + \vert u \vert ^{p-2}u = D_{x}^{-2}\triangle_{y}u,\quad u \neq0, u\in Y_{1}. $$

Define on $Y_{1}$ the following functionals:

$$\begin{aligned} &L_{1}(u) = \int \biggl({1\over 2}u_{x}^{2} + {1\over 2} \bigl\vert D_{x}^{-1} \nabla_{y}u \bigr\vert ^{2} + {1\over 2} \vert u \vert ^{2}- {1\over p} \vert u \vert ^{p} \biggr)\,dV\quad\hbox{and} \\ &I_{1}(u) = \int \bigl(u_{x}^{2} + \bigl\vert D_{x}^{-1}\nabla_{y}u \bigr\vert ^{2} + \vert u \vert ^{2} - \vert u \vert ^{p} \bigr)\,dV. \end{aligned}$$

Set

$$\Gamma_{1} = \bigl\{ u\in Y_{1}: u \neq0, I_{1}(u) = 0 \bigr\} \quad \hbox{and}\quad d_{1} = \inf _{u\in\Gamma_{1}}L_{1}(u). $$

Then according to the inequality (1.5), both $L_{1}$ and $I_{1}$ are well defined and $C^{1}$ on $Y_{1}$. The following definition is by now standard.

#### Definition 2.1

An element $v\in Y_{1}$ is said to be a solution of (2.1) if and only if *v* is a critical point of $L_{1}$, i.e., $L_{1}'(v) = 0$. Moreover, $v\in Y_{1}$ is said to be a minimal action solution of (2.1) if $v\neq0$, $L_{1}'(v) = 0$ and $L_{1}(v) \leq L_{1}(u)$ for any $u\in\Gamma_{1}$.

The following lemmas will play important roles in what follows.

#### Lemma 2.2

*For any* $u\in Y_{1}$ *and* $u\neq0$, *there is a unique* $s_{u} > 0$ *such that* $s_{u} u\in\Gamma_{1}$. *Moreover*, *if* $I_{1}(u) < 0$ *then* $0 < s_{u} < 1$.

#### Proof

For $u\neq0$ and $s > 0$, we have

$$I_{1}(s u) = \int \bigl(s^{2}u_{x}^{2} + s^{2} \bigl\vert D_{x}^{-1}\nabla_{y}u \bigr\vert ^{2} + s^{2} \vert u \vert ^{2}- s^{p} \vert u \vert ^{p} \bigr)\,dV. $$

Hence from direct computations, we get

$$s_{u} = \bigl( \Vert u \Vert _{Y_{1}}^{2} \bigr)^{1\over {p-2}} \biggl( \int \vert u \vert ^{p}\,dV \biggr)^{-{1\over {p-2}}}. $$

Clearly from the expression of $I_{1}(u)$, we know that if $I_{1}(u) < 0$, then $\|u\|_{Y_{1}}^{2} < \int|u|^{p}\,dV$ and therefore $0 < s_{u} < 1$. □

#### Lemma 2.3

*The set* $\Gamma_{1}$ *is a manifold and there exists* $\rho> 0$ *such that*, *for any* $u\in\Gamma_{1}$, $\|u\|_{Y_{1}} \geq\rho> 0$.

#### Proof

Firstly, it is observed from Lemma 2.2 that $\Gamma _{1}\neq\emptyset$. For any $u\in\Gamma_{1}$,

$$\bigl\langle I_{1}'(u),u\bigr\rangle _{Y_{1}} = 2 \Vert u \Vert ^{2}_{Y_{1}} - p \int \vert u \vert ^{p}\,dV = (2 - p ) \int \vert u \vert ^{p}\,dV < 0. $$

Hence $\Gamma_{1}$ is a manifold. Secondly, for any $u\in\Gamma_{1}$, using inequality (1.5) and Young inequality, we know that there is a positive constant *C* such that

$$\Vert u \Vert ^{2}_{Y_{1}} = \int \vert u \vert ^{p}\,dV \leq C \Vert u \Vert ^{p}_{Y_{1}}. $$

It is deduced that $\|u\|_{Y_{1}} \geq C^{-{1\over {p-2}}} := \rho> 0$. The proof is complete. □

#### Lemma 2.4

*If* $v \in\Gamma_{1}$ *and* $L_{1}(v) = d_{1}$, *then* *v* *is a critical point of* $L_{1}$ *on* $Y_{1}$, *i*.*e*. $L_{1}'(v) = 0$.

#### Proof

By Lagrangian multiplier rule, there is $\theta\in\mathbb {R}$ such that $L_{1}'(v) = \theta I_{1}'(v)$. Note that $\langle L_{1}'(v),v\rangle_{Y_{1}} = I_{1}(v) = 0$ and

$$\bigl\langle I_{1}'(v),v\bigr\rangle _{Y_{1}} = (2 - p ) \int \vert u \vert ^{p}\,dV < 0. $$

One easily obtains $\theta= 0$. Therefore $L_{1}'(v) = 0$. □

#### Theorem 2.5

*We see that* $d_{1} > 0$ *and there is a* $\phi\in\Gamma_{1}$ *such that* $d_{1} = L_{1}(\phi)$. *Moreover*, *ϕ* *is a minimal action solution of* (2.1).

#### Proof

It is easy to see from Lemma 2.3 that $d_{1} > 0$. According to Definition 2.1 and Lemma 2.4, we only need to prove that there is $\phi\in\Gamma_{1}$ such that $d_{1} = L_{1}(\phi)$.

Let $\{u_{n}\}_{n\in\mathbb{N}} \subset\Gamma_{1}$ be a minimizing sequence of $d_{1}$, i.e. $u_{n} \neq0$, $I_{1}(u_{n}) = 0$ and $d_{1} + o(1) = L_{1}(u_{n})$. By $I_{1}(u_{n}) = 0$ and the anisotropic Sobolev inequality (1.5), we know that $\|u_{n}\|_{Y_{1}}$ is bounded. Moreover, Lemma 2.3 implies that $\|u_{n}\|_{Y_{1}}$ is uniformly bounded away from zero and we see that

$$\liminf_{n\to\infty} \int \vert u_{n} \vert ^{p}\,dV = \liminf _{n\to\infty} \Vert u_{n} \Vert _{Y_{1}}^{2} > 0. $$

Note that, for any $V \equiv(x,y)\in\mathbb{R}^{N}$,

$$\begin{aligned} &L_{1}\bigl(u(\cdot+x,\cdot+y_{1}, \ldots, \cdot+y_{N-1})\bigr) = L_{1}\bigl(u(\cdot)\bigr)\quad \hbox{and} \\ &I_{1}\bigl(u(\cdot+x,\cdot+y_{1}, \ldots, \cdot+y_{N-1})\bigr) = I_{1}\bigl(u(\cdot)\bigr). \end{aligned}$$

We see from the concentration compactness lemma of Lions [8] that there are $V^{n} \equiv(x^{n}, y^{n})\in\mathbb{R}^{N}$, where $y^{n} = (y_{1}^{n}, \ldots, y_{N-1}^{n})$, such that

$$\varphi_{n}(x,y):= u_{n}\bigl(x+x^{n}, y_{1}+y^{n}_{1}, \ldots, y_{N-1}+y^{n}_{N-1} \bigr) $$

satisfies

$$L_{1}(\varphi_{n}) = L_{1}(u_{n})\quad \hbox{and}\quad I_{1}(\varphi_{n}) = I_{1}(u_{n}) = 0. $$

Moreover, there is $\phi\in Y_{1}$ and $\phi\neq0$ such that $\phi_{n} \rightharpoonup \phi$ weakly in $Y_{1}$ and $\varphi_{n} \to\phi$ a.e. in $\mathbb{R}^{N}$.

If $I_{1}(\phi) < 0$, then by Lemma 2.2 there is a $0 < s_{\phi}< 1$ such that $s_{\phi}\phi\in\Gamma_{1}$. Therefore using the Fatou lemma and the fact that $I_{1}(\varphi_{n}) = 0$, we obtain

$$\begin{aligned} d_{1} + o(1) &= L_{1}(\varphi_{n}) = \biggl( {1\over 2}-{1\over p} \biggr) \int \vert \varphi_{n} \vert ^{p}\,dV \geq \biggl({1\over 2}-{1\over p} \biggr) \int \vert \phi \vert ^{p}\,dV + o(1) \\ &= \biggl({1\over 2}-{1\over p} \biggr)s_{\phi}^{-p} \int \vert s_{\phi}\phi \vert ^{p}\,dV + o(1) = s_{\phi}^{-p}L_{1}(s_{\phi}\phi) + o(1) \end{aligned}$$

as *n* large enough. It is deduced from $0 < s_{\phi}< 1$ that $d_{1} > L_{1}(s_{\phi}\phi)$ which is a contradiction because of $s_{\phi}\phi\in\Gamma_{1}$.

If $I_{1}(\phi) > 0$, then using Brezis–Lieb lemma [2] one has

$$0 = I_{1}(\varphi_{n}) = I_{1}(\phi) + I_{1}(v_{n}) + o(1), $$

where $\varphi_{n} - \phi:= v_{n}$ in the remaining part of this section. $I_{1}(\phi) > 0$ implies that

2.2 $$ \limsup_{n\to\infty} I_{1}(v_{n}) < 0. $$

From Lemma 2.2 we know that there are $s_{n} := s_{v_{n}}$ such that $s_{n}v_{n}\in\Gamma_{1}$. Moreover, we claim that $\limsup_{n\to\infty}s_{n} \in(0,1)$. Indeed if $\limsup_{n\to\infty}s_{n} = 1$, then there is a subsequence $\{s_{n_{k}}\}$ such that $\lim_{k\to\infty} s_{n_{k}} = 1$. Therefore from $s_{n_{k}}v_{n_{k}} \in\Gamma_{1}$, one has

$$I_{1}(v_{n_{k}}) = I_{1}(s_{n_{k}}v_{n_{k}}) + o(1) = o(1). $$

This contradicts (2.2). Hence $\limsup_{n\to\infty}s_{n} \in (0,1)$. Since, for *n* large enough,

$$\begin{aligned} d_{1} + o(1) &= \biggl({1\over 2}-{1\over p} \biggr) \int \vert \varphi_{n} \vert ^{p}\,dV \geq \biggl({1\over 2}-{1\over p} \biggr) \int \vert v_{n} \vert ^{p}\,dV + o(1) \\ &\geq \biggl({1\over 2}-{1\over p} \biggr)s_{n}^{-p} \int \vert s_{n}v_{n} \vert ^{p}\,dV + o(1) = s_{n}^{-p}L_{1}(s_{n}v_{n}) + o(1), \end{aligned}$$

one has $d_{1} > L_{1}(s_{n}v_{n})$, which is a contradiction because $s_{n}v_{n}\in\Gamma_{1}$.

Thus $I_{1}(\phi) = 0$. Next we claim that $\|v_{n}\|_{Y_{1}}\to0$ as $n\to\infty$. We prove this claim by a contradiction. If $\|v_{n}\| _{Y_{1}}\not\to 0$ as $n\to\infty$, then there is a subsequence $\{v_{n_{k}}\}_{k\in\mathbb{N}}\subset\{v_{n}\}_{n\in\mathbb{N}}$, such that $\|v_{n_{k}}\|_{Y_{1}}\to C > 0$ as $k\to\infty$. Using Brezis–Lieb lemma [2], one has

$$0 = I_{1}(\varphi_{n_{k}}) = I_{1}(v_{n_{k}}) + I_{1}(\phi) + o(1). $$

Hence $I_{1}(v_{n_{k}}) = o(1)$. According to Lemma 2.2, there are $\rho_{n_{k}} > 0$ such that $I_{1}(\rho_{n_{k}}v_{n_{k}}) = 0$. Moreover, $\rho_{n_{k}}\to1$ as $k \to\infty$. Using Brezis–Lieb lemma [2] again, we see that, as *n* grows large enough,

$$\begin{aligned} d_{1} + o(1) &= L_{1}(\varphi_{n_{k}}) = L_{1}(v_{n_{k}}) + L_{1}(\phi) + o(1) \\ &= L_{1}(\rho_{n_{k}}v_{n_{k}}) + L_{1}( \phi) + o(1) \\ &\geq d_{1} + d_{1} + o(1), \end{aligned}$$

which is impossible because of $d_{1} > 0$. Hence we have proven that $\|v_{n_{k}}\|_{Y_{1}} \to0$ as $k\to\infty$. Therefore $L_{1}(\varphi_{n_{k}}) \to L_{1}(\phi)$ and $d_{1} = L_{1}(\phi)$. □

Next, we give some properties of the minimal action solution *ϕ* obtained above. These properties seem to be of independent interests and will be very useful in what follows.

#### Lemma 2.6

*Let* *ϕ* *be a minimal action solution of* (2.1). *Then* $I_{1}(\phi) = 0$,

$$\begin{aligned} &Q_{1}(\phi) := \int \biggl(\phi_{x}^{2} + \bigl\vert D_{x}^{-1}\nabla_{y} \phi \bigr\vert ^{2} - {{(2N-1)(p-2)}\over {2p}} \vert \phi \vert ^{p} \biggr)\,dV = 0\quad\textit{and} \\ &R_{1}(\phi) := \int \biggl(\phi_{x}^{2} - {{N(p-2)}\over {2p}} \vert \phi \vert ^{p} \biggr)\,dV = 0. \end{aligned}$$

*Moreover*, *we see that*

2.3 $$ \begin{aligned} & \int \bigl\vert D_{x}^{-1}\nabla_{y} \phi \bigr\vert ^{2}\,dV = {{N-1}\over N} \int\phi_{x}^{2}\, dV, \\ & \int \vert \phi \vert ^{p}\,dV = {{2p}\over {N(p-2)}} \int\phi_{x}^{2}\,dV\quad\textit{and} \\ & \int \vert \phi \vert ^{2}\,dV = {{(3-2N)p + 2(2N-1)}\over {N(p-2)}} \int\phi_{x}^{2}\,dV. \end{aligned} $$

#### Proof

Since *ϕ* is a minimal action solution of (2.1), $L_{1}'(\phi) = 0$. First, we define

$$\phi_{\delta}(x,y) = \delta^{\frac{N}{2}}\phi(\delta x, \delta y),\quad y = (y_{1}, \ldots, y_{N-1})\in\mathbb{R}^{N-1}. $$

Then, by direct computation, we see that

$$\begin{aligned} &\int (\partial_{x}\phi_{\delta})^{2} \,dV = \delta^{2} \int\phi_{x}^{2} \,dV;\qquad \int \vert \phi_{\delta} \vert ^{p} \,dV= \delta^{\frac{N(p-2)}{2}} \int \vert \phi \vert ^{p} \,dV; \\ &\int \bigl\vert D_{x}^{-1}\nabla_{y} \phi_{\delta}\bigr\vert ^{2}\,dV = \int \bigl\vert D_{x}^{-1}\nabla_{y} \phi \bigr\vert ^{2}\,dV\quad\hbox{and}\quad \int \vert \phi_{\delta} \vert ^{2}\,dV= \int \vert \phi \vert ^{2}\,dV. \end{aligned}$$

Hence

$$L_{1}(\phi_{\delta}) = \frac{\delta^{2}}{2} \int\phi_{x}^{2} \,dV + {1\over 2} \int \bigl\vert D_{x}^{-1}\nabla_{y} \phi \bigr\vert ^{2}\,dV + {1\over 2} \int \vert \phi \vert ^{2}\,dV - \frac {\delta^{\frac{N(p-2)}{2}}}{p} \int \vert \phi \vert ^{p} \,dV. $$

Therefore

$$R_{1}(\phi) = \frac{\partial L_{1}(\phi_{\delta})}{\partial\delta}\biggm|_{\delta=1}=\biggl\langle L_{1}'(\phi), \frac{\partial\phi_{\delta}}{\partial \delta}\biggm|_{\delta=1}\biggr\rangle _{Y_{1}} = 0. $$

Next, we define

$$\phi^{\delta}(x,y) = \delta^{\frac{2N-1}{2}}\phi\bigl(\delta x, \delta^{2} y\bigr),\quad y = (y_{1}, \ldots, y_{N-1})\in\mathbb{R}^{N-1}. $$

Then, by direct computation, we obtain

$$\begin{aligned} &\int \bigl(\partial_{x}\phi^{\delta}\bigr)^{2} \,dV = \delta^{2} \int\phi_{x}^{2} \,dV;\qquad \int \bigl\vert \phi^{\delta}\bigr\vert ^{p} \,dV= \delta^{\frac{(2N-1)(p-2)}{2}} \int \vert \phi \vert ^{p} \,dV; \\ &\int \bigl\vert D_{x}^{-1}\nabla_{y} \phi^{\delta}\bigr\vert ^{2}\,dV = \delta^{2} \int \bigl\vert D_{x}^{-1}\nabla _{y} \phi \bigr\vert ^{2}\,dV\quad\hbox{and}\quad \int \bigl\vert \phi^{\delta}\bigr\vert ^{2}\,dV= \int \vert \phi \vert ^{2}\,dV. \end{aligned}$$

Hence

$$L_{1}\bigl(\phi^{\delta}\bigr) = \frac{\delta^{2}}{2} \int \bigl(\phi_{x}^{2} + \bigl\vert D_{x}^{-1}\nabla_{y}\phi \bigr\vert ^{2} \bigr) \,dV + \frac{1}{2} \int \vert \phi \vert ^{2}\,dV - \frac{\delta^{\frac {(2N-1)(p-2)}{2}}}{p} \int \vert \phi \vert ^{p} \,dV. $$

Therefore

$$Q_{1}(\phi) = \frac{\partial L_{1}(\phi^{\delta})}{\partial\delta}\biggm|_{\delta=1}=\biggl\langle L_{1}'(\phi), \frac{\partial\phi^{\delta}}{\partial \delta}\biggm|_{\delta=1}\biggr\rangle _{Y_{1}} = 0. $$

The proof is complete. □

#### Remark

From Lemma 2.6, one also obtains

2.4 $$ \begin{aligned} & \int \bigl\vert D_{x}^{-1}\nabla_{y} \phi \bigr\vert ^{2}\,dV = {{(N-1)(p-2)}\over {(3-2N)p + 2(2N-1)}} \int\phi^{2}\,dV, \\ & \int \vert \phi \vert ^{p}\,dV = {{2p}\over {(3-2N)p + 2(2N-1)}} \int\phi^{2}\,dV\quad\hbox{and} \\ & \int\phi_{x}^{2}\,dV = {{N(p-2)}\over {(3-2N)p + 2(2N-1)}} \int\phi^{2}\,dV. \end{aligned} $$

### Another characterization of the minimal action solutions

In this subsection, we give another characterization of the minimal action solution *ϕ* of (2.1) obtained in the previous subsection. We emphasize that this characterization will play a key role in the process of estimating *α*. Define

$$F(u)= \int_{\mathbb{R}^{N}} \bigl(u_{x}^{2} + \bigl\vert D_{x}^{-1}\nabla_{y} u \bigr\vert ^{2} + u^{2} \bigr)\,dV, \quad u\in Y_{1} $$

and for $r > 0$ set

$$F_{r} = \inf \biggl\{ F(u) : u\in Y_{1}\mbox{ and } \int \vert u \vert ^{p}\,dV = r \biggr\} . $$

Then we have the following proposition.

#### Proposition 2.7

*Let* *ϕ* *be a minimal action solution of* (2.1). *Then* *ϕ* *is a minimizer of* $F_{r}$ *with* $r = \int|\phi|^{p}\,dV$.

#### Proof

Since *ϕ* is a minimal action solution of (2.1), we know that $L_{1}(\phi) \leq L_{1}(u)$ for any $u\in Y_{1}$ with $u\neq0$ and $I_{1}(u) = 0$. Denote

$$F_{r_{0}} = \inf \biggl\{ F(u) : u\in Y_{1}\mbox{ and } \int \vert u \vert ^{p}\,dV = \int \vert \phi \vert ^{p}\,dV \biggr\} . $$

One immediately has $F(\phi) \geq F_{r_{0}}$.

Next, we will prove that, for any $u\in Y_{1}$ with $\int|u|^{p}\,dV = \int|\phi|^{p}\,dV$,

$$F(\phi) \leq F(u). $$

For any $\mu> 0$, $I_{1}(\mu u) = \mu^{2}F(u) - \mu^{p}\int|u|^{p}\,dV$. Hence

$$\mu_{0} = \bigl(F(u) \bigr)^{1\over {p-2}} \biggl( \int \vert u \vert ^{p}\,dV \biggr)^{-{1\over {p-2}}} $$

is such that $I_{1}(\mu_{0} u) = 0$. The fact that $\mu_{0} u\neq0$ implies that

$$\begin{aligned} L_{1}(\phi)&\leq L_{1}(\mu_{0}u) = {1\over 2}\mu_{0}^{2}F(u) - {1\over p}\mu_{0}^{p} \int \vert u \vert ^{p}\,dV \\ &= \biggl({1\over 2} - {1\over p} \biggr) \bigl(F(u) \bigr)^{p\over {p-2}} \biggl( \int \vert u \vert ^{p}\,dV \biggr)^{-{2\over {p-2}}} \\ &= \biggl({1\over 2} - {1\over p} \biggr) \bigl(F(u) \bigr)^{p\over {p-2}} \biggl( \int \vert \phi \vert ^{p}\,dV \biggr)^{-{2\over {p-2}}}. \end{aligned}$$

Since $\int|\phi|^{p}\,dV = F(\phi)$ and $L_{1}(\phi) = ({1\over 2} - {1\over p})F(\phi)$, one deduces that

$$\bigl(F(\phi)\bigr)^{p\over {p-2}} \leq \bigl(F(u)\bigr)^{p\over {p-2}}, $$

which implies that $F(\phi) \leq F(u)$. Since *u* is chosen arbitrarily, one has $F(\phi) \leq F_{r_{0}}$. Combining this with $F(\phi) \geq F_{r_{0}}$, one has $F(\phi) = F_{r_{0}}$ and hence *ϕ* is a minimizer of $F_{r}$ with $r = \int|\phi|^{p}\,dV$. □

### Estimate of the smallest constant *α*

In this subsection, we estimate the constant *α* of (1.5). To simplify the notation, we denote $T = (3-2N)p + 2(2N-1)$. Consider the following minimization problem:

2.5 $$ \alpha^{-1} = \inf_{u\neq0, u\in Y_{1}}J_{1}(u), $$

where

$$J_{1}(u) = \biggl( \int \vert u \vert ^{2}\,dV \biggr)^{T\over 4} \biggl( \int u_{x}^{2}\,dV \biggr)^{{N(p-2)}\over 4} \prod _{k=1}^{N-1} \biggl( \int \bigl\vert D_{x}^{-1}\partial_{y_{k}}u \bigr\vert ^{2}\,dV \biggr)^{{p-2}\over 4} \biggl( \int \vert u \vert ^{p}\,dV \biggr)^{-1}. $$

We have the following theorem.

#### Theorem 2.8

*Let* $2 < p < p_{*}$ *and* $T = (3-2N)p + 2(2N-1)$. *Then*

B $$\begin{aligned} \begin{aligned}[b] \alpha^{-1} &= \biggl({T\over {2p}} \biggr)^{T\over 4} \biggl({{N^{N}(p-2)^{2N-1}}\over {(2p)^{2N-3}T^{2}}} \biggr)^{{p-2}\over 4} \biggl( \int \vert \phi \vert ^{2}\,dV \biggr)^{{p-2}\over 2} \\ &= \biggl({T\over {2p}} \biggr)^{T\over 4} \biggl( {{N^{N}(p-2)^{2N-1}}\over {(2p)^{2N-3}T^{2}}} \biggr)^{{p-2}\over 4} \biggl({T\over {p-2}}d_{1} \biggr)^{{p-2}\over 2}, \end{aligned} \end{aligned}$$

*where* *ϕ* *is the minimal action solution of* (2.1) *obtained in the above and* $d_{1} = L_{1}(\phi)$.

#### Remark

From Theorem 2.8, we know that $\alpha^{-1}$ can be exactly expressed by *N*, *p* and the minimal action solution *ϕ* of (2.1). Even though we do not know if the minimal action solution of (2.1) is unique or not, the second equality of (B) implies that $\alpha^{-1}$ is independent of the choice of the minimal action solution *ϕ*.

#### Proof of Theorem 2.8

The proof is divided into three steps. In the first two steps, we prove the first equality of (B). In the third step, we prove the second equality of (B).

*Step 1.* In this step, we prove that

$$\alpha^{-1}\geq \biggl({T\over {2p}} \biggr)^{T\over 4} \biggl({{N^{N}(p-2)^{2N-1}}\over {(2p)^{2N-3}T^{2}}} \biggr)^{{p-2}\over 4} \biggl( \int \vert \phi \vert ^{2}\,dV \biggr)^{{p-2}\over 2}. $$

For any $u\in Y_{1}$ and $u\neq0$, denote $u \equiv u(x, y_{1}, \ldots, y_{N-1})$. We define

$$w(x,y) = \lambda u(\mu x, \xi_{1}y_{1}, \ldots, \xi_{N-1}y_{N-1}), $$

where *λ*, *μ*, $\xi_{1}, \ldots, \xi_{N-1}$ are $N+1$ positive parameters which will be determined later. From direct computation, one obtains

2.6 $$\begin{aligned} \begin{aligned} &\int \bigl\vert D_{x}^{-1}\partial_{y_{k}}w \bigr\vert ^{2}\,dV = \lambda^{2} \mu^{-3} \biggl(\prod_{j\neq k}\xi_{j}^{-1} \biggr)\xi_{k} \int \bigl\vert D_{x}^{-1}\partial_{y_{k}}u \bigr\vert ^{2}\,dV,\quad k=1, \ldots, N-1; \\ &\int \vert w \vert ^{p}\,dV = \lambda^{p} \mu^{-1} \Biggl(\prod_{k=1}^{N-1} \xi_{k}^{-1} \Biggr) \int \vert u \vert ^{p}\,dV; \\ &\int w_{x}^{2}\,dV = \lambda^{2} \mu \Biggl(\prod_{k=1}^{N-1}\xi_{k}^{-1} \Biggr) \int u_{x}^{2}\,dV; \\ &\int w^{2}\,dV = \lambda^{2}\mu^{-1} \Biggl(\prod_{k=1}^{N-1}\xi_{k}^{-1} \Biggr) \int u^{2}\,dV. \end{aligned} \end{aligned}$$

Here *λ*, *μ*, $\xi_{1}, \ldots, \xi_{N-1}$ are determined by the following $N + 1$ equations:

2.7 $$ \begin{aligned}[b] &\lambda^{2} \mu^{-3} \biggl(\prod _{j\neq k}\xi_{j}^{-1} \biggr) \xi_{k} \int \bigl\vert D_{x}^{-1}\partial_{y_{k}}u \bigr\vert ^{2}\,dV \\ &\quad = {{p-2}\over {(3-2N)p + 2(2N-1)}} \int \phi^{2}\,dV, \end{aligned} $$

where for $k=1, \ldots, N-1$, (2.7) is denoted by (2.7)_*k*_;

2.8 $$\begin{aligned} &\lambda^{p} \mu^{-1} \Biggl(\prod _{k=1}^{N-1}\xi_{k}^{-1} \Biggr) \int \vert u \vert ^{p}\,dV = {{2p}\over {(3-2N)p + 2(2N-1)}} \int\phi^{2}\,dV, \end{aligned}$$

2.9 $$\begin{aligned} &\lambda^{2} \mu \Biggl(\prod _{k=1}^{N-1}\xi_{k}^{-1} \Biggr) \int u_{x}^{2}\,dV ={{N(p-2)}\over {(3-2N)p + 2(2N-1)}} \int\phi^{2}\,dV. \end{aligned}$$

Next, we solve *λ*, *μ*, $\xi_{1}, \ldots, \xi_{N-1}$ from (2.7)_*k*_, where $k = 1, \ldots, N-1$, (2.8) and (2.9). Firstly, (2.8) and (2.9) imply that

2.10 $$ \mu^{2} = {{N(p-2)}\over {2p}}\lambda^{p-2} \int \vert u \vert ^{p}\,dV \biggl( \int u_{x}^{2}\,dV \biggr)^{-1}. $$

Using (2.8) and (2.7)_*k*_, one gets

2.11 $$ \lambda^{p-2}\mu^{2}\xi_{k}^{-2} \int \vert u \vert ^{p}\,dV = {{2p}\over {p-2}} \int \bigl\vert D_{x}^{-1}\partial_{y_{k}}u \bigr\vert ^{2}\,dV, \quad k=1, \ldots, N-1. $$

It is now deduced from (2.10) and (2.11) that

2.12 $$ \begin{aligned}[b] &\lambda^{2(p-2)} \biggl( \int \vert u \vert ^{p}\,dV \biggr)^{2} \biggl( \int u_{x}^{2}\,dV \biggr)^{-1} \biggl( \int \bigl\vert D_{x}^{-1}\partial_{y_{k}}u \bigr\vert ^{2}\,dV \biggr)^{-1}\xi_{k}^{-2}\\ &\quad = {1\over N} \biggl({{2p}\over {p-2}} \biggr)^{2}. \end{aligned} $$

Using (2.9), (2.10) and (2.12), we obtain

$$\lambda^{4}\mu^{2}\prod_{k=1}^{N-1} \xi_{k}^{-2} \biggl( \int u_{x}^{2}\,dV \biggr)^{2} = \biggl( {{N(p-2)}\over {(3-2N)p + 2(2N-1)}} \int \phi^{2}\,dV \biggr)^{2}. $$

Hence

$$\begin{aligned} & \lambda^{4+(p-2)(3-2N)} \biggl( \int u_{x}^{2}\,dV \biggr)^{N} \biggl( \int \vert u \vert ^{p}\,dV \biggr)^{3-2N}\prod _{k=1}^{N-1} \int \bigl\vert D_{x}^{-1}\partial_{y_{k}}u \bigr\vert ^{2}\,dV \\ &\quad = {{N^{N}(p-2)^{2}}\over {((3-2N)p + 2(2N-1))^{2}}} \biggl({{p-2}\over {2p}} \biggr)^{2N-3} \biggl( \int \phi^{2}\,dV \biggr)^{2}. \end{aligned}$$

Secondly, remember the notation $T=(3-2N)p + 2(2N-1)$, the above equality can be written as

$$\begin{aligned} & \lambda^{T} \biggl( \int u_{x}^{2}\,dV \biggr)^{N} \biggl( \int \vert u \vert ^{p}\,dV \biggr)^{3-2N}\prod _{k=1}^{N-1} \int \bigl\vert D_{x}^{-1}\partial_{y_{k}}u \bigr\vert ^{2}\,dV \\ &\quad = {{N^{N}(p-2)^{2N-1}}\over {(2p)^{2N-3}T^{2}}} \biggl( \int \phi^{2}\,dV \biggr)^{2}. \end{aligned}$$

Therefore

2.13 $$ \begin{aligned}[b] \lambda={}& \biggl( {{N^{N}(p-2)^{2N-1}}\over {(2p)^{2N-3}T^{2}}} \biggr)^{1\over T} \biggl( \int\phi^{2}\,dV \biggr)^{2\over T} \biggl( \int u_{x}^{2}\,dV \biggr)^{{-N}\over T} \\ &{}\times \biggl( \int \vert u \vert ^{p}\,dV \biggr)^{{2N-3}\over T}\prod _{k=1}^{N-1} \biggl( \int \bigl\vert D_{x}^{-1}\partial_{y_{k}}u \bigr\vert ^{2}\,dV \biggr)^{{-1}\over T}. \end{aligned} $$

It is deduced from (2.6), (2.8) and (2.13) that

$$\begin{aligned} \int w^{2}\,dV ={}& {{2p}\over T} \lambda^{2} \int u^{2}\,dV \biggl(\lambda^{p} \int \vert u \vert ^{p}\,dV \biggr)^{-1} \int \vert \phi \vert ^{2}\,dV \\ ={}& {{2p}\over T} \int \vert \phi \vert ^{2}\,dV \int u^{2}\,dV \biggl( \int \vert u \vert ^{p}\,dV \biggr)^{-1} \lambda^{2-p} \\ ={}& {{2p}\over T} \biggl({{N^{N}(p-2)^{2N-1}}\over {(2p)^{2N-3}T^{2}}} \biggr)^{{2-p}\over T} \biggl( \int \vert \phi \vert ^{2}\,dV \biggr)^{{2(2N-1)-(2N-1)p}\over T} \\ &{} \times \biggl( \int u_{x}^{2}\,dV \biggr)^{{-N(2-p)}\over T} \int u^{2}\,dV \biggl( \int \vert u \vert ^{p}\,dV \biggr)^{{-4}\over T}\prod _{k=1}^{N-1} \biggl( \int \bigl\vert D_{x}^{-1}\partial_{y_{k}}u \bigr\vert ^{2}\,dV \biggr)^{{p-2}\over T}. \end{aligned}$$

According to Proposition 2.7, *ϕ* is a minimizer of $F_{r_{0}}$ with $r_{0} = \int|\phi|^{p}\,dV$. Hence we obtain from $\int|w|^{p}\,dV = \int|\phi|^{p}\,dV$ $F(w) \geq F(\phi)$. Using the definition of *w* and (2.4), one immediately has

$$\int w^{2}\,dV \geq \int\phi^{2}\,dV. $$

It is deduced that

$$\begin{aligned} & \biggl( \int u_{x}^{2}\,dV \biggr)^{{-N(2-p)}\over T} \int u^{2}\,dV \biggl( \int \vert u \vert ^{p}\,dV \biggr)^{{-4}\over T}\prod _{k=1}^{N-1} \biggl( \int \bigl\vert D_{x}^{-1}\partial_{y_{k}}u \bigr\vert ^{2}\,dV \biggr)^{{p-2}\over T} \\ &\quad \geq {T\over {2p}} \biggl({{N^{N}(p-2)^{2N-1}}\over {(2p)^{2N-3}T^{2}}} \biggr)^{{p-2}\over T} \biggl( \int \vert \phi \vert ^{2}\,dV \biggr)^{1-{{2(2N-1)-(2N-1)p}\over T}}. \end{aligned}$$

Therefore

$$J_{1}(u) \geq \biggl({T\over {2p}} \biggr)^{T\over 4} \biggl({{N^{N}(p-2)^{2N-1}}\over {(2p)^{2N-3}T^{2}}} \biggr)^{{p-2}\over 4} \biggl( \int \vert \phi \vert ^{2}\,dV \biggr)^{{p-2}\over 2}. $$

Since *u* is arbitrary, we deduce that

$$\alpha^{-1} \geq \biggl({T\over {2p}} \biggr)^{T\over 4} \biggl({{N^{N}(p-2)^{2N-1}}\over {(2p)^{2N-3}T^{2}}} \biggr)^{{p-2}\over 4} \biggl( \int \vert \phi \vert ^{2}\,dV \biggr)^{{p-2}\over 2}. $$

*Step 2.* We prove that

$$\alpha^{-1} \leq \biggl({T\over {2p}} \biggr)^{T\over 4} \biggl({{N^{N}(p-2)^{2N-1}}\over {(2p)^{2N-3}T^{2}}} \biggr)^{{p-2}\over 4} \biggl( \int \vert \phi \vert ^{2}\,dV \biggr)^{{p-2}\over 2}. $$

In the first place, using mean value inequality, one has

$$\begin{aligned} \prod_{k=1}^{N-1} \biggl( \int \bigl\vert D_{x}^{-1}\partial_{y_{k}} \phi \bigr\vert ^{2}\,dV \biggr)^{{p-2}\over 4} &= \Biggl(\prod _{k=1}^{N-1} \int \bigl\vert D_{x}^{-1}\partial_{y_{k}} \phi \bigr\vert ^{2}\,dV \Biggr)^{{p-2}\over 4} \\ &\leq \Biggl[ \Biggl({1\over {N-1}}\sum_{k=1}^{N-1} \int \bigl\vert D_{x}^{-1}\partial_{y_{k}} \phi \bigr\vert ^{2}\,dV \Biggr)^{N-1} \Biggr]^{{p-2}\over 4}\\ &= \biggl({{p-2}\over T} \int\phi^{2}\,dV \biggr)^{{(N-1)(p-2)}\over 4}. \end{aligned}$$

In the second place, using the fact that $\phi\neq0$, $\phi\in Y_{1}$ and (2.4), one obtains immediately

$$\begin{aligned} J_{1}(\phi) ={}& \biggl( \int\phi^{2}\,dV \biggr)^{T\over 4} \biggl( \int\phi_{x}^{2}\,dV \biggr)^{{N(p-2)}\over 4} \\ &{}\times \biggl( \int \vert \phi \vert ^{p}\,dV \biggr)^{-1} \prod _{k=1}^{N-1} \biggl( \int \bigl\vert D_{x}^{-1}\partial_{y_{k}} \phi \bigr\vert ^{2}\,dV \biggr)^{{p-2}\over 4} \\ \leq {}& \biggl( \int\phi^{2}\,dV \biggr)^{T\over 4} \biggl( {{N(p-2)}\over T} \int\phi^{2}\,dV \biggr)^{{N(p-2)}\over 4} \\ &{}\times \biggl({{2p}\over T} \int \vert \phi \vert ^{2}\,dV \biggr)^{-1} \biggl({{p-2}\over T} \int \phi^{2}\,dV \biggr)^{{(N-1)(p-2)}\over 4} \\ ={}& \biggl({T\over {2p}} \biggr)^{T\over 4} \biggl( {{N^{N}(p-2)^{2N-1}}\over {(2p)^{2N-3}T^{2}}} \biggr)^{{p-2}\over 4} \biggl( \int \vert \phi \vert ^{2}\,dV \biggr)^{{p-2}\over 2}. \end{aligned}$$

From *Step 1.* and *Step 2.*, we get the first equality of (B).

*Step 3.* Now we prove the second equality of (B). Since $d_{1} = L_{1}(\phi)$, we obtain from (2.4) that

$$\begin{aligned} d_{1} &= \int \biggl({1\over 2}\phi_{x}^{2} + {1\over 2}\phi^{2} +{1\over 2} \bigl\vert D_{x}^{-1}\nabla_{y_{k}}\phi \bigr\vert ^{2} - {1\over p} \vert \phi \vert ^{p} \biggr)\, dV \\ &= {1\over 2} \biggl({{N(p-2)}\over T} + {{(N-1)(p-2)}\over T} + {1\over 2} \biggr) \int \vert \phi \vert ^{2}\,dV - {1\over p} \cdot{{2p}\over T} \int \vert \phi \vert ^{2}\,dV \\ &= {{p-2}\over T} \int \vert \phi \vert ^{2}\,dV. \end{aligned}$$

Combining this with the first equality of (B), one gets the second equality of (B). □

## Estimate of the smallest constant *β*

In this section, we study the smallest constant *β* in (1.6). We use variational methods and the ideas from the previous section. Observing the proofs in the previous section, we find that it is very important to do the scaling and solve *λ*, *μ* and $\xi_{k}$, where $k=1, 2, \ldots, N-1$, from $N + 1$ equations; see (2.7)_*k*_, (2.8) and (2.9). However, as we will see below, in the process of estimating *β*, we still need to solve $N + 1$ positive parameters, but we only have *N* equations; see (3.7) and (3.6)_*k*_, where $k=1, 2, \ldots, N-1$. Hence we need to do the scaling and investigate the parameters carefully. Keeping the notation $p_{*}$ in mind, we consider the following minimization problem:

3.1 $$ \beta^{-1} = \inf_{u\neq0, u\in Y_{0}}J_{0}(u), $$

where

$$J_{0}(u) = \biggl( \int u_{x}^{2}\,dV \biggr)^{N\over {2N-3}} \prod _{k=1}^{N-1} \biggl( \int \bigl\vert D_{x}^{-1}\partial_{y_{k}}u \bigr\vert ^{2}\,dV \biggr)^{1\over {2N-3}} \biggl( \int \vert u \vert ^{p_{*}}\,dV \biggr)^{-1} $$

and $Y_{0}$ is the closure of $\partial_{x}(C_{0}^{\infty}(\mathbb{R}^{N}))$ under the norm

$$\Vert u \Vert ^{2}_{Y_{0}} := \int_{\mathbb{R}^{N}} \bigl(u_{x}^{2} + \bigl\vert D_{x}^{-1}\nabla_{y} u \bigr\vert ^{2} \bigr)\,dV. $$

The following related equation is useful in what follows:

3.2 $$ u_{xx} + \vert u \vert ^{p_{*}-2}u = D_{x}^{-2}\triangle_{y}u,\quad u \neq0, u \in Y_{0}. $$

Define on $Y_{0}$ the functionals

$$\begin{aligned} &L_{0}(u) = \int \biggl({1\over 2}u_{x}^{2} + {1\over 2} \bigl\vert D_{x}^{-1} \nabla_{y}u \bigr\vert ^{2} - {1\over {p_{*}}} \vert u \vert ^{p_{*}} \biggr)\,dV\quad\hbox{and} \\ &I_{0}(u) = \int \bigl(u_{x}^{2} + \bigl\vert D_{x}^{-1}\nabla_{y}u \bigr\vert ^{2} - \vert u \vert ^{p_{*}} \bigr)\,dV. \end{aligned}$$

Set

$$\Gamma_{0} = \bigl\{ u\in Y_{0}: u \neq0, I_{0}(u) = 0 \bigr\} \quad \hbox{and}\quad d_{0} = \inf _{u\in\Gamma_{0}}L_{0}(u). $$

Then according to inequality (1.6), both $L_{0}$ and $I_{0}$ are well defined and $C^{1}$ on $Y_{0}$.

### Definition 3.1

An element $v\in Y_{0}$ is said to be a solution of (3.2) if and only if *v* is a critical point of $L_{0}$, i.e., $L_{0}'(v) = 0$. Moreover, $v\in Y_{0}$ is said to be a minimal action solution of (3.2) if $v\neq0$, $L_{0}'(v) = 0$ and $L_{0}(v) \leq L_{0}(u)$ for any $u\in\Gamma_{0}$.

### Lemma 3.2

*For any* $u\in Y_{0}$ *and* $u\neq0$, *there is a unique* $s_{u} > 0$ *such that* $s_{u} u\in\Gamma_{0}$. *Moreover*, *if* $I_{0}(u) < 0$ *then* $0 < s_{u} < 1$.

### Lemma 3.3

*The set* $\Gamma_{0}$ *is a manifold and there exists* $\rho> 0$ *such that*, *for any* $u\in\Gamma_{0}$, $\|u\|_{Y_{0}} \geq\rho> 0$.

### Lemma 3.4

*If* $v \in\Gamma_{0}$ *and* $L_{0}(v) = d_{0}$, *then* *v* *is a critical point of* $L_{0}$ *on* $Y_{0}$, *i*.*e*. $L_{0}'(v) = 0$.

### Remark

The proofs of Lemma 3.2, Lemma 3.3 and Lemma 3.4 are similar to the proofs of Lemma 2.2, Lemma 2.3 and Lemma 2.4. We omit the details here.

### Theorem 3.5

*We see that* $d_{0} > 0$ *and there is a* $\psi\in\Gamma_{0}$ *such that* $d_{0} = L_{0}(\psi)$. *Moreover*, *ψ* *is a minimal action solution of* (3.2).

### Remark

The proof of Theorem 3.5 follows lines similar to the proof of Theorem 2.5. We emphasize that in the proof of Theorem 2.5, the functionals $L_{1}$ and $I_{1}$ only have invariance under translations, i.e., for any $V\in \mathbb{R}^{N}$, $L_{1}(u(\cdot+V)) = L_{1}(u(\cdot))$ and $I_{1}(u(\cdot+V)) = I_{1}(u(\cdot))$. But, in the case $p = p_{*}$, the functionals $L_{0}$ and $I_{0}$ not only have invariance under translation, but also have invariance under dilation; see below $(IUD)$ for details. Hence, we give a detailed proof of Theorem 3.5.

### Proof of Theorem 3.5

By Lemma 3.3 we know that $d_{0} > 0$. According to Definition 3.1 and Lemma 3.4, we only need to prove that there is $\psi\in\Gamma_{0}$ such that $d_{0} = L_{0}(\psi)$.

Let $\{u_{n}\}_{n\in\mathbb{N}}\subset\Gamma_{0}$ be a minimizing sequence of $d_{0}$, i.e., $u_{n} \neq0$, $I_{0}(u_{n}) = 0$ and $d_{0} + o(1) = L_{0}(u_{n})$ for *n* large enough. Then it is easy to see from $I_{0}(u_{n}) = 0$ that $\|u_{n}\|_{Y_{0}}$ is uniformly bounded with respect to *n*. Moreover, Lemma 3.3 implies that $\|u_{n}\|_{Y_{0}}$ is uniformly bounded away from zero and we see that

$$\liminf_{n\to\infty} \int \vert u_{n} \vert ^{p_{*}}\,dV = \liminf _{n\to\infty} \Vert u_{n} \Vert _{Y_{0}}^{2} > 0. $$

Note that, for any $V \equiv(x,y_{1}, \ldots, y_{N-1})\in \mathbb{R}^{N}$,

$$\begin{aligned} &L_{0}\bigl(u(\cdot+x,\cdot+y_{1}, \ldots, \cdot+y_{N-1})\bigr) = L_{0}\bigl(u(\cdot)\bigr)\quad \hbox{and} \\ &I_{0}\bigl(u(\cdot+x,\cdot+y_{1}, \ldots, \cdot+y_{N-1})\bigr) = I_{0}\bigl(u(\cdot)\bigr). \end{aligned}$$

Moreover, for any $\lambda > 0$, denoting

$$u^{\lambda}(x,y) := \lambda u\bigl(\lambda^{2\over {2N-3}}x, \lambda^{4\over {2N-3}}y_{1}, \ldots, \lambda^{4\over {2N-3}}y_{N-1} \bigr), $$

we have

IUD $$\begin{aligned} L_{0}\bigl(u^{\lambda}\bigr) = L_{0}(u) \quad\hbox{and}\quad I_{0}\bigl(u^{\lambda}\bigr) = I_{0}(u). \end{aligned}$$

We obtain from the concentration compactness lemma of Lions [8] that there are $\gamma_{n}$ and $V^{n} \equiv(x^{n}, y_{1}^{n}, \ldots, y_{N-1}^{n})\in\mathbb{R}^{N}$ such that $\psi_{n}(x,y):= \gamma_{n} u_{n}(\gamma^{2\over {2N-3}}_{n}(x+x^{n}), \gamma^{4\over {2N-3}}_{n}(y_{1}+y^{n}_{1}), \ldots, \gamma^{4\over {2N-3}}_{n}(y_{N-1}+y^{n}_{N-1}))$ satisfies

$$L_{0}(\psi_{n}) = L_{0}(u_{n})\quad \hbox{and}\quad I_{0}(\psi_{n}) = I_{0}(u_{n}). $$

Additionally, there is $\psi\in Y_{0}$ such that $\psi_{n} \rightharpoonup\psi$ weakly in $Y_{0}$, $\psi_{n} \to\psi$ a.e. in $\mathbb{R}^{N}$ and $\psi\neq0$.

If $I_{0}(\psi) < 0$, then by Lemma 3.2 there is a $s_{\psi}$ such that $0 < s_{\psi}< 1$ and $s_{\psi}\psi\in\Gamma_{0}$. Therefore using the Fatou lemma and $I_{0}(\psi_{n}) = I_{0}(u_{n}) = 0$, we obtain

$$\begin{aligned} d_{0} + o(1) &= L_{0}(\varphi_{n}) = \biggl( {1\over 2}-{1\over {p_{*}}} \biggr) \int \vert \psi_{n} \vert ^{p_{*}}\,dV \geq {1\over {2N-1}} \int \vert \psi \vert ^{p_{*}}\,dV + o(1) \\ &= {1\over {2N-1}}s_{\psi}^{-p_{*}} \int \vert s_{\psi}\psi \vert ^{p_{*}}\,dV + o(1) = s_{\psi}^{-{p_{*}}}L_{0}(s_{\psi}\psi) + o(1). \end{aligned}$$

It is now deduced from $0 < s_{\psi}< 1$ that $d_{0} > L_{0}(s_{\psi}\psi)$, which is a contradiction because of $s_{\psi}\psi\in \Gamma_{0}$.

If $I_{0}(\psi) > 0$, then using the Brezis–Lieb lemma [2] one has

$$0 = I_{0}(\psi_{n}) = I_{0}(\psi) + I_{0}(v_{n}) + o(1), $$

where we denote $\psi_{n} - \psi$ by $v_{n}$ in the rest of this section. This and $I_{0}(\psi) > 0$ imply that

3.3 $$ \limsup_{n\to\infty} I_{0}(v_{n}) < 0. $$

From Lemma 3.2 we know that there are $s_{n} := s_{v_{n}}$ such that $s_{n}v_{n}\in\Gamma_{0}$. Moreover, we claim that $\limsup_{n\to\infty}s_{n} \in(0,1)$. Indeed if $\limsup_{n\to\infty}s_{n} = 1$, then there is a subsequence $\{s_{n_{k}}\}$ such that $\lim_{k\to\infty} s_{n_{k}} = 1$. Therefore from $s_{n_{k}}v_{n_{k}} \in\Gamma_{0}$ one has

$$I_{0}(v_{n_{k}}) = I_{0}(s_{n_{k}}v_{n_{k}}) + o(1) = o(1). $$

This contradicts (3.3). Hence $\limsup_{n\to\infty}s_{n} \in (0,1)$. Since

$$\begin{aligned} d_{0} + o(1) &= \biggl({1\over 2}-{1\over {p_{*}}} \biggr) \int \vert \psi _{n} \vert ^{p_{*}}\,dV \\ &\geq {1\over {2N-1}} \int \vert v_{n} \vert ^{p_{*}}\,dV + o(1) \\ &= {1\over {2N-1}}s_{n}^{-p_{*}} \int \vert s_{n}v_{n} \vert ^{p_{*}}\,dV + o(1) \\ & = s_{n}^{-p_{*}}L_{0}(s_{n}v_{n}) + o(1), \end{aligned}$$

one deduces that $d_{0} > L_{0}(s_{n}v_{n})$, which is a contradiction because of $s_{n}v_{n}\in\Gamma_{0}$.

Thus $I_{0}(\phi) = 0$. Now similar to the proof of Theorem 2.5, we obtain $\|v_{n_{k}}\|_{Y_{0}}\to0$ as $k\to \infty$. Therefore $L_{0}(\psi_{n_{k}}) \to L_{0}(\psi)$ and $d_{0} = L_{0}(\psi)$. □

Next we give some properties of the minimal action solution *ψ* of (3.2).

### Lemma 3.6

*Let* *ψ* *be a minimal action solution of* (3.2). *Then* $I_{0}(\phi) = 0$ *and*

$$R_{0}(\phi) := \int \biggl(\psi_{x}^{2} - {N\over {2N-1}} \vert \psi \vert ^{p_{*}} \biggr)\,dV = 0. $$

*Moreover*, *we see that*

3.4 $$ \begin{aligned} & \int \bigl\vert D_{x}^{-1}\nabla_{y} \phi \bigr\vert ^{2}\,dV = {{N-1}\over N} \int\phi_{x}^{2}\, dV\quad\textit{and} \\ & \int \vert \phi \vert ^{p_{*}}\,dV = {{2N-1}\over N} \int\phi_{x}^{2}\,dV. \end{aligned} $$

### Proof

The proof is similar to Lemma 2.6. We omit the details here. □

Next, we give another characterization of the minimal action solutions *ψ* of (3.2). For $u\in Y_{0}$, define

$$K(u)= \int \bigl(u_{x}^{2} + \bigl\vert D_{x}^{-1}\nabla_{y} u \bigr\vert ^{2} \bigr)\,dV $$

and for $r > 0$ set

$$K_{r} = \inf \biggl\{ K(u) : u\in Y_{0} \mbox{ and } \int \vert u \vert ^{p_{*}}\, dV = r \biggr\} . $$

Then we have the following proposition.

### Proposition 3.7

*Let* *ψ* *be a minimal action solution of* (3.2). *Then* *ψ* *is a minimizer of* $K_{r}$ *with* $r = \int|\psi|^{p_{*}}\,dV$.

### Proof

The proof is similar to Proposition 2.7. We omit the details. □

Now we are in a position to study the smallest constant *β* in (1.6).

### Theorem 3.8

*Let* *ψ* *be the minimal action solution of* (3.2) *obtained in Theorem* 3.5 *and* $d_{0} = L_{0}(\psi)$. *The smallest constant* *β* *in* (1.6) *is*

$$\begin{aligned} \beta^{-1} &= (2N-1)^{-1}N^{{N-2}\over {2N-3}} \biggl( \int\psi_{x}^{2}\,dV \biggr)^{2\over {2N-3}} \\ &= (2N-1)^{-1}N^{N\over {2N-3}}d_{0}^{2\over {2N-3}}. \end{aligned}$$

### Remark

From Theorem 3.8, *β* is unique, since the minimum $d_{0}$ is unique. We point out that $\beta^{-1}$ is independent of the choice of the minimal action solution *ψ* of (3.2), although we do not know the uniqueness of the minimal action solution. In fact the uniqueness of the minimal action solution of (3.2) was an open problem.

### Proof of Theorem 3.8

The proof is divided into three steps.

*Step 1.* In this step, we prove that

$$\beta^{-1}\geq (2N-1)^{-1}N^{{N-2}\over {2N-3}} \biggl( \int\psi_{x}^{2}\, dV \biggr)^{2\over {2N-3}}. $$

For any $u\in Y_{0}$ and $u\neq0$, denote $u \equiv u(x,y)$ with $y = (y_{1}, \ldots, y_{N-1})$. Define

$$w(x,y) = \lambda u(\mu x, \xi_{1}y_{1}, \ldots, \xi_{N-1}y_{N-1}), $$

where *λ*, *μ*, $\xi_{1}, \ldots, \xi_{N-1}$ are positive parameters which will be determined later. Then, by direct computations, we see that

3.5 $$ \begin{aligned} &\int \bigl\vert D_{x}^{-1}\partial_{y_{k}}w \bigr\vert ^{2}\,dV = \lambda^{2} \mu^{-3} \biggl(\prod_{j\neq k}\xi_{j}^{-1} \biggr)\xi_{k} \int \bigl\vert D_{x}^{-1}\partial_{y_{k}}u \bigr\vert ^{2}\,dV,\quad k=1, \ldots, N-1; \\ &\int \vert w \vert ^{p_{*}}\,dV = \lambda^{p_{*}} \mu^{-1} \Biggl(\prod_{k=1}^{N-1} \xi_{k}^{-1} \Biggr) \int \vert u \vert ^{p_{*}}\,dV; \\ &\int w_{x}^{2}\,dV = \lambda^{2} \mu \Biggl(\prod_{k=1}^{N-1}\xi_{k}^{-1} \Biggr) \int u_{x}^{2}\,dV. \end{aligned} $$

In here *λ*, *μ*, $\xi_{1}, \ldots, \xi_{N-1}$ are determined by the following *N* equations:

3.6 $$ \lambda^{2} \mu^{-3} \biggl(\prod _{j\neq k}\xi_{j}^{-1} \biggr) \xi_{k} \int \bigl\vert D_{x}^{-1}\partial_{y_{k}}u \bigr\vert ^{2}\,dV = {1\over N} \int\psi_{x}^{2}\,dV, $$

where for $k=1, \ldots, N-1$, (3.6) is denoted by (3.6)_*k*_;

3.7 $$ \lambda^{p_{*}} \mu^{-1} \Biggl(\prod _{k=1}^{N-1}\xi_{k}^{-1} \Biggr) \int \vert u \vert ^{p_{*}}\,dV = {{2N-1}\over N} \int\psi_{x}^{2}\,dV. $$

(*Note:* we need to solve $N+1$ variables only from *N* equations.)

In the first place, from (3.6)_1_ and (3.7), one gets

3.8 $$ \lambda^{2-p_{*}} \mu^{-2}\xi_{1}^{2} \int \bigl\vert D_{x}^{-1}\partial_{y_{1}}u \bigr\vert ^{2}\,dV ={1\over {2N-1}} \int \vert u \vert ^{p_{*}}\,dV. $$

In the second place, from (3.6)_*k*_, one gets

$$\xi_{k}^{-1} = \biggl( \int \bigl\vert D_{x}^{-1}\partial_{y_{k}}u \bigr\vert ^{2}\,dV \biggr)^{1\over 2} \biggl( \int \bigl\vert D_{x}^{-1}\partial_{y_{1}}u \bigr\vert ^{2}\,dV \biggr)^{-{1\over 2}}\xi_{1}^{-1}, \quad k=2, \ldots, N-1. $$

It is now deduced from (3.6)_1_ and the expression of $\xi_{k}^{-1}$ ( $k=2, \ldots, N-1$) that

3.9 $$ \lambda^{2} \mu^{-3}\xi_{1}^{3-N} \biggl( \int \bigl\vert D_{x}^{-1}\partial_{y_{1}}u \bigr\vert ^{2}\,dV \biggr)^{2-{N\over 2}} \prod _{k=2}^{N-1} \biggl( \int \bigl\vert D_{x}^{-1}\partial_{y_{k}}u \bigr\vert ^{2}\,dV \biggr)^{1\over 2}={1\over N} \int\phi_{x}^{2}\,dV. $$

Equations (3.8) and (3.9) imply that

3.10 $$ \begin{aligned}[b] & \lambda^{3(2-p_{*})-4} \xi_{1}^{6-2(3-N)} \biggl( \int \bigl\vert D_{x}^{-1}\partial_{y_{1}}u \bigr\vert ^{2}\,dV \biggr)^{3-(4-N)} \prod _{k=2}^{N-1} \biggl( \int \bigl\vert D_{x}^{-1}\partial_{y_{k}}u \bigr\vert ^{2}\,dV \biggr)^{-1} \\ &\quad = N^{2} \biggl({1\over {2N-1}} \biggr)^{3} \biggl( \int \vert u \vert ^{p_{*}}\,dV \biggr)^{3} \biggl( \int \psi_{x}^{2}\,dV \biggr)^{-2}. \end{aligned} $$

Combining this with the fact of $p_{*}=2(2N-1)/(2N-3)$, one obtains

3.11 $$ \begin{aligned}[b] & \lambda^{{-8N}\over {2N-3}} \xi_{1}^{2N} \biggl( \int \bigl\vert D_{x}^{-1}\partial_{y_{1}}u \bigr\vert ^{2}\,dV \biggr)^{N-1} \prod _{k=2}^{N-1} \biggl( \int \bigl\vert D_{x}^{-1}\partial_{y_{k}}u \bigr\vert ^{2}\,dV \biggr)^{-1} \\ &\quad = {{N^{2}}\over {(2N-1)^{3}}} \biggl( \int \vert u \vert ^{p_{*}}\,dV \biggr)^{3} \biggl( \int \psi_{x}^{2}\,dV \biggr)^{-2}. \end{aligned} $$

Note that (3.8) can be written as

3.12 $$ \lambda^{-{{8N}\over {2N-3}}} \mu^{-4N}\xi_{1}^{4N} \biggl( \int \bigl\vert D_{x}^{-1}\partial_{y_{1}}u \bigr\vert ^{2}\,dV \biggr)^{2N} ={1\over {(2N-1)^{2N}}} \biggl( \int \vert u \vert ^{p_{*}}\,dV \biggr)^{2N}. $$

We obtain from (3.11) and (3.12)

3.13 $$ \begin{aligned}[b] & \mu^{4N} \xi_{1}^{-2N} \biggl( \int \bigl\vert D_{x}^{-1}\partial_{y_{1}}u \bigr\vert ^{2}\,dV \biggr)^{-N-1}\prod _{k=2}^{N-1} \biggl( \int \bigl\vert D_{x}^{-1}\partial_{y_{k}}u \bigr\vert ^{2}\,dV \biggr)^{-1} \\ &\quad= N^{2}(2N-1)^{2N-3} \biggl( \int \vert u \vert ^{p_{*}}\,dV \biggr)^{3-2N} \biggl( \int\psi_{x}^{2}\,dV \biggr)^{-2}. \end{aligned} $$

Therefore

3.14 $$ \begin{aligned}[b] \mu^{4} \xi_{1}^{-2}={}& N^{2\over N}(2N-1)^{{2N-3}\over N} \biggl( \int \vert u \vert ^{p_{*}}\,dV \biggr)^{{3-2N}\over N} \biggl( \int\psi_{x}^{2}\,dV \biggr)^{{-2}\over N} \\ & {}\times \biggl( \int \bigl\vert D_{x}^{-1}\partial_{y_{1}}u \bigr\vert ^{2}\,dV \biggr)^{{N+1}\over N}\prod _{k=2}^{N-1} \biggl( \int \bigl\vert D_{x}^{-1}\partial_{y_{k}}u \bigr\vert ^{2}\,dV \biggr)^{1\over N}. \end{aligned} $$

Writing (3.12) as

$$\lambda^{-{4\over {2N-3}}} = {1\over {(2N-1)}}\mu^{2} \xi_{1}^{-2} \int \vert u \vert ^{p_{*}}\,dV \biggl( \int \bigl\vert D_{x}^{-1}\partial_{y_{1}}u \bigr\vert ^{2}\,dV \biggr)^{-1}, $$

we deduce from (3.7) and (3.5) that

$$\begin{aligned} \int w_{x}^{2}\,dV ={}& \lambda^{2}\mu\prod _{k=1}^{N-1}\xi_{k}^{-1} \int u_{x}^{2}\,dV \\ ={}& \lambda^{2}\mu \int u_{x}^{2}\,dV \biggl[{{2N-1}\over N} \int\psi_{x}^{2}\,dV\lambda^{-p_{*}}\mu \biggl( \int \vert u \vert ^{p_{*}}\,dV \biggr)^{-1} \biggr] \\ ={}& \lambda^{2-p_{*}}\mu^{2} \int u_{x}^{2}\,dV \biggl( \int \vert u \vert ^{p_{*}}\,dV \biggr)^{-1} {{2N-1}\over N} \int\psi_{x}^{2}\,dV\quad\bigl(\hbox{using (3.8)}\bigr) \\ ={}& \mu^{4}\xi_{1}^{-2}{1\over N} \int\psi_{x}^{2}\,dV \int u_{x}^{2}\,dV \biggl( \int \bigl\vert D_{x}^{-1}\partial_{y_{1}}u \bigr\vert ^{2}\,dV \biggr)^{-1} \\ ={}& {1\over N} \int\psi_{x}^{2}\,dV \int u_{x}^{2}\,dV \biggl( \int \bigl\vert D_{x}^{-1}\partial_{y_{1}}u \bigr\vert ^{2}\,dV \biggr)^{-1} \\ &{}\times N^{2\over N}(2N-1)^{{2N-3}\over N} \biggl( \int \vert u \vert ^{p_{*}}\,dV \biggr)^{{3-2N}\over N} \biggl( \int\psi_{x}^{2}\,dV \biggr)^{{-2}\over N} \\ &{}\times \biggl( \int \bigl\vert D_{x}^{-1}\partial_{y_{1}}u \bigr\vert ^{2}\,dV \biggr)^{{N+1}\over N}\prod _{k=2}^{N-1} \biggl( \int \bigl\vert D_{x}^{-1}\partial_{y_{k}}u \bigr\vert ^{2}\,dV \biggr)^{1\over N} \\ ={}& N^{{2\over N}-1}(2N-1)^{{2N-3}\over N} \biggl( \int\psi_{x}^{2}\,dV \biggr)^{1-{2\over N}} \int u_{x}^{2}\,dV \\ &{}\times\prod_{k=1}^{N-1} \biggl( \int \bigl\vert D_{x}^{-1}\partial_{y_{k}}u \bigr\vert ^{2}\,dV \biggr)^{1\over N} \biggl( \int \vert u \vert ^{p_{*}}\,dV \biggr)^{{3-2N}\over N}. \end{aligned}$$

From the definition of *w* and the fact that *ψ* is a minimizer of $K_{r}$ with $r = \int|\psi|^{p_{*}}\,dV$, we get

$$\int w_{x}^{2}\,dV \geq \int\psi_{x}^{2}\,dV, $$

which implies that

$$\begin{aligned} & \int u_{x}^{2}\,dV \biggl( \int \vert u \vert ^{p}\,dV \biggr)^{{3-2N}\over N}\prod _{k=1}^{N-1} \biggl( \int \bigl\vert D_{x}^{-1}\partial_{y_{k}}u \bigr\vert ^{2}\,dV \biggr)^{1\over N} \\ &\quad\geq N^{1-{2\over N}}(2N-1)^{{2N-3}\over N} \biggl( \int\psi_{x}^{2}\,dV \biggr)^{2\over N}. \end{aligned}$$

Therefore

$$J_{0}(u) \geq N^{{N-2}\over {2N-3}}(2N-1)^{-1} \biggl( \int \psi_{x}^{2}\,dV \biggr)^{2\over {2N-3}}. $$

Since $u\neq0$ and $u\in Y_{0}$ is chosen arbitrarily, we get

$$\beta^{-1} \geq(2N-1)^{-1}N^{{N-2}\over {2N-3}} \biggl( \int\psi_{x}^{2}\, dV \biggr)^{2\over {2N-3}}. $$

*Step 2.* In this step, we prove that

$$\beta^{-1} \leq(2N-1)^{-1}N^{{N-2}\over {2N-3}} \biggl( \int\psi_{x}^{2}\, dV \biggr)^{2\over {2N-3}}. $$

Since $\psi\neq0$ and $\psi\in Y_{0}$, we obtain from Lemma 3.6 and the mean value inequality that

$$\begin{aligned} J_{0}(\psi) &= \biggl( \int\psi_{x}^{2}\,dV \biggr)^{N\over {2N-3}} \biggl( \int \vert \psi \vert ^{p_{*}}\,dV \biggr)^{-1} \prod_{k=1}^{N-1} \biggl( \int \bigl\vert D_{x}^{-1}\partial_{y_{k}} \psi \bigr\vert ^{2}\,dV \biggr)^{1\over {2N-3}} \\ & \leq \biggl( \int\psi_{x}^{2}\,dV \biggr)^{N\over {2N-3}} \biggl( {{2N-1}\over N} \int\psi_{x}^{2}\,dV \biggr)^{-1} \Biggl( {1\over {N-1}}\sum_{k=1}^{N-1} \int \bigl\vert D_{x}^{-1}\partial_{y_{k}} \psi \bigr\vert ^{2}\,dV \Biggr)^{{N-1}\over {2N-3}} \\ &= \biggl( \int\psi_{x}^{2}\,dV \biggr)^{N\over {2N-3}} \biggl( {{2N-1}\over N} \int\psi_{x}^{2}\,dV \biggr)^{-1} \biggl( {1\over N} \int\psi_{x}^{2}\,dV \biggr)^{{N-1}\over {2N-3}} \\ &= (2N-1)^{-1}N^{{N-2}\over {2N-3}} \biggl( \int\psi_{x}^{2}\,dV \biggr)^{2\over {2N-3}}. \end{aligned}$$

Therefore

$$\beta^{-1} \leq(2N-1)^{-1}N^{{N-2}\over {2N-3}} \biggl( \int\psi_{x}^{2}\, dV \biggr)^{2\over {2N-3}}. $$

Combining *Step 1.* and *Step 2.*, we get $\beta^{-1}$ as stated in the theorem.

*Step 3.* We prove that

$$\beta^{-1} = (2N-1)^{-1}N^{N\over {2N-3}}d_{0}^{2\over {2N-3}}. $$

Indeed, since $d_{0} = L_{0}(\psi)$, we obtain from Lemma 3.6 that

$$\begin{aligned} d_{0} &= \int \biggl({1\over 2}\psi_{x}^{2}\,dV + {1\over 2} \bigl\vert D_{x}^{-1} \nabla_{y_{k}}\psi \bigr\vert ^{2} - {1\over {p_{*}}} \vert \phi \vert ^{p_{*}} \biggr)\,dV \\ &= \biggl({1\over 2} - {1\over {p_{*}}}\cdot {{2N-1}\over N} + {1\over 2}\cdot{{N-1}\over N} \biggr) \int\psi_{x}^{2}\,dV \\ &= {1\over N} \int\psi_{x}^{2}\,dV. \end{aligned}$$

Combining this with the first equality of $\beta^{-1}$ in the statement of Theorem 3.8, we get the second equality of $\beta^{-1}$ in Theorem 3.8. □

## Conclusions

In this paper, we not only estimated the smallest constant in a general *N*-dimensional anisotropic Sobolev inequality in the subcritical case; we also gave an estimate of the smallest constant for *N*-dimensional anisotropic Sobolev inequality in the critical case.
